# Identification of key performance indicators for on-farm animal welfare incidents: possible tools for early warning and prevention

**DOI:** 10.1186/2046-0481-64-13

**Published:** 2011-10-07

**Authors:** Patricia C Kelly, Simon J More, Martin Blake, Alison J Hanlon

**Affiliations:** 1 Department of Agriculture, Fisheries and Food, Agriculture House, Kildare Street, Dublin 2, Ireland; 2 Veterinary Sciences Centre, University College Dublin, Belfield, Dublin 4, Ireland

## Abstract

**Background:**

The objective of this study was to describe aspects of case study herds investigated by the Department of Agriculture, Fisheries and Food (DAFF) in which animal welfare incidents occurred and to identify key performance indicators (KPIs) that can be monitored to enhance the Early Warning System (EWS). Despite an EWS being in place for a number of years, animal welfare incidents continue to occur. Questionnaires regarding welfare incidents were sent to Superintending Veterinary Inspectors (SVIs), resulting in 18 herds being chosen as case study herds, 12 of which had a clearly defined welfare incident date. For each study herd, data on six potential KPIs were extracted from DAFF databases. The KPIs for those herds with a clearly defined welfare incident date were studied for a consecutive four year window, with the fourth year being the 'incident year', when the welfare incident was disclosed. For study herds without a clearly defined welfare incident date, the KPIs were determined on a yearly basis between 2001 and 2009.

**Results:**

We found that the late registration of calves, the use of on-farm burial as a method of carcase disposal, an increasing number of moves to knackeries over time and records of animals moved to 'herd unknown' were notable on the case farms.

**Conclusion:**

Four KPIs were prominent on the case study farms and warrant further investigation in control herds to determine their potential to provide a framework for refining current systems of early warning and prevention.

## Background

The Early Warning System (EWS) was established in Ireland in 2004 when the Minister for Agriculture and Food accepted the recommendation of the Farm Animal Welfare Advisory Council (FAWAC) for the establishment of a collaborative, nationwide early warning/intervention system for farm animal welfare cases. The EWS currently involves a partnership between the Department of Agriculture, Fisheries and Food (DAFF), the Irish Farmers Association (IFA), the Irish Society for the Prevention of Cruelty to Animals (ISPCA) and the Health Service Executive (HSE), and aims to identify and address cases in which the welfare of farm animals is compromised. Its purpose is to provide a framework within which problems can be identified and resolved before they become critical or overwhelming.

The assessment and monitoring of farm animal welfare are key objectives of the European Community's Action Plan on Animal Welfare (2008) [1]. However, there is an inconsistent approach to the recording of on-farm animal welfare across the community [2]. Developing a harmonised European monitoring system to assess the quality of farming systems and their impact on the diseases and welfare of animals is advocated by the European Food Safety Authority [2]. In Ireland, the Animal Field Inspection Testing database (AFIT) was introduced in 2008, to collate information on regulatory inspections performed by Veterinary Inspectors (VIs). Technical Agricultural Officers and District Superintendents may also be involved in inspections to herds that are considered as 'at risk' herds. Inspection reports contain data on farm animal welfare and other regulatory issues such as animal remedies, marts, and transport. Welfare inspections are carried out specifically for calves, pigs, laying hens (cages, barn and free-range) and, most recently, broilers. In addition, general inspections covering all animal species kept on a farm (including adult cattle, sheep, horses etc.) are also carried out. The inspections cover information on types of animals kept, animal health, staffing levels, stocking density, record keeping, housing, feed and water. Other EU countries, including the UK and Sweden, also maintain databases on animal welfare. The UK's database contains records of inspections carried out on farms and includes details of the types and numbers of animals inspected and any action taken. In Sweden, the database contains information on all persons and businesses that are subject to animal welfare control.

Despite this, farm animal welfare incidents remain an ongoing concern in Ireland. Although the EWS has been established for some years, no quantitative evaluation has yet been conducted to evaluate its effectiveness. An unpublished study was conducted by DAFF between September 2006 and March 2007, before the introduction of AFIT, to investigate animal welfare incidents in Ireland (Pat Flanagan: An investigation into On-Farm Welfare Incidents, 2007). It provided baseline data on the number and category of dead and moribund animals recorded in animal welfare incidents, the number of animals seized and disposed of and explored the human resource implications for DAFF. The study reported that a total of 494 animal welfare incidents were investigated by DVO staff between 1 September 2006 and 31 July 2007. In the study, in excess of 1,500 animals were found dead during on-farm investigations, 78 animals required euthanasia on farm and 619 had to be seized and disposed off. Seventeen percent of herd owners had animal identification problems. It highlighted the need for further research to better understand the aetiology of farm animal incidents, identify and understand risk factors associated with on-farm animal welfare incidents in Ireland and thereby provide opportunities to refine current systems of early warning and prevention.

This study will form part of a broader study which will include comparisons with control herds and a separate study investigating the human factors that influence on-farm animal welfare incidents. The objectives of this current study are to describe aspects of case study herds investigated by DAFF in which animal welfare incidents occurred and to identify potential key performance indicators (KPIs) which will later be studied in control herds to see if future monitoring of these KPIs can enhance the EWS.

## Materials and methods

### 2.1 Case study herd enrolment

In 2009, Senior Superintending Veterinary Inspectors (SSVI) and Superintending Veterinary Inspectors (SVI) from DAFF Divisional Veterinary Offices (DVOs) were sent screening questionnaires to identify herds in their region with reported farm animal welfare incidents. For the purpose of this research, a farm animal welfare incident was defined as 'any situation where a person in charge of cattle or sheep causes avoidable pain or suffering to those cattle or sheep, or fails to take steps to prevent avoidable pain or suffering to cattle or sheep under his or her care, or fails to respond expeditiously to cattle or sheep that are experiencing avoidable pain or suffering under his or her care'. A case study herd was defined as any herd where a farm animal welfare incident was reported. From this initial list of herds, subsequent selection of case study herds was undertaken by three of the authors, with consideration given to species (cattle, sheep), geographic spread, farm size, the welfare issue, and the likelihood of the herd keeper cooperating with an interview, to facilitate a later study to investigate if any life-events influenced management of the herd. As the majority of cases involved cattle, it was decided to limit the study to cattle.

### 2.2 Data collection

For each study herd, data were collected about the welfare incident, including the welfare issue, the animal species and the date/duration of the problem. General data about each farm were extracted from two DAFF national databases: the Animal Identification & Movement database (AIM) and the Animal Health Computer System (AHCS). AIM records the sex of the animal, date of birth, herd movements during its lifetime and the final status in Ireland e.g. disposal through a 'knackery' or by 'on-farm burial', slaughter in a 'factory' for an animal destined for the food chain, 'export centres' for animals that are exported to other countries and 'location unknown' for animals whose whereabouts cannot be satisfactorily explained. AHCS records information on the tuberculosis (TB) and brucellosis status of herds, the type of herd, the names and contact details of the herd keeper, the private veterinary practitioner and the attendant Veterinary Inspector (VI).

The following data were collected from AIM and AHCS for each study herd:

a. General data

• the sex and age of the herd keeper,

• the type of herd (dairy or beef),

• other species kept on the holding (if any), and

• the size of the holding, cattle numbers per hectare.

b. Yearly livestock data

• total number of cattle in herd on 1 January each year, with subsets based on class - cows, heifers, males,

• the number of calves born during the course of each year, including their dates of birth and the dates that they were registered on the database by the herd keeper, and

• the number of all animal exits recorded each year, including to knackeries, by on-farm burial, to market, to slaughter and unknown locations.

### 2.3 Data management and analysis

The identity of each study herd was anonymised (designated A to R).

Six potential KPIs were identified from the yearly livestock data, based on a previous DAFF unpublished welfare study (Pat Flanagan: An investigation into On-Farm Welfare Incidents, 2007), the experience of the authors and from discussions with VIs heavily involved in welfare cases. These included:

• the number of cattle in the herd,

• the number of cows that gave birth to a calf each year,

• the number of late registrations (greater than 27 days after birth) in the herd,

• the percentage of all cattle exits from the herd that were moved to a knackery,

• the percentage of all cattle exits from the herd that were disposed of by on-farm burial, and

• the percentage of animals recorded as being moved to 'herd unknown'

For each study herd with a clearly defined welfare incident, a consecutive four year window ('the study period') was identified, with the fourth year being the 'incident year', when the welfare incident was disclosed. The three year retrospective period was selected based on the authors' experience of welfare cases, data trends and the experience of VIs considered experts in dealing with welfare cases. The number of cattle in the herd on 1 January each year, by animal class (cows, heifers, males), was extracted from AIM. A female animal was considered a cow if 30 months of age or older, and a heifer otherwise. It was not possible to distinguish bulls from bullocks, so all male animals were referred to as males.

For each year of the study period, we then calculated:

• the number of late calf registrations (greater than 27 days after birth),

• the percentage of cattle exits separately attributed to (a) movement to a knackery, (b) disposal through on-farm burial, or (c) moved to a herd described as 'herd unknown', and

• the number of cows that gave birth to a calf each year (indicating the number of breeding females available to the number of calves actually registered).

For study herds without a clearly defined welfare incident (long-term welfare incidents), each of the above-mentioned calculations was determined on a yearly basis between 2001 and 2009.

Data were managed using Microsoft Excel (Microsoft Corporation, Redmond, WA, USA).

## Results

Twenty-one replies to the screening questionnaire were received from 15 DVOs (a response rate of 53.5%).

### 3.1 Demographics

Eighteen case herds were enrolled, including 12 single farms and three sets of two associated farms i.e. herds H and I, K and L, Q and R (Table 1). Welfare issues were longstanding in nature in six of the herds (D, F, G, N, Q and R). In all 18 herds, the number of cattle per hectare ranged from 0.32 to 2.82 (median 1.31) at the time of the study, but no information was available on the quality of the land or feeding systems on the farms. Farm size ranged from 30.9 to 390 hectares, with an average size of 108.3 ha and a median of 63 ha. Four of the herds were registered to female herd owners, one to a married couple and the remaining 13 to men. Age range of herd keepers varied from 31 to 84 years. In two cases, the age of the herd keeper was not known. The median age of the herd keepers whose age was known, was 50 years at the time of the incident. The average age was 55 years.

**Table 1:** Description of the 18 case herds, their keepers and welfare problems

Herd ID	Age of herd owner (years) at the time of the incident	Sex of herd owner	Herd type	Other species	Size of farm (hectares)	Welfare problem (as described by investigation VI)
A^1^	65	M	Suckler	No	30.9	Registration issues and high mortality

B^2^	40	M	Dairy	No	63	Registration issues and poor conditions

C^3^	46	M	Dairy	No	84.2	High mortality associated with neglected and starving cattle

D	Not recorded	F	Suckler	Horses, poultry, sheep	63.1	High mortality and unburied sheep carcases

E	Not recorded	M	Suckler	No	56	Dead cattle found in yard

F	63	M	Suckler	Horses	125.4	History of problems with cattle and horses

G	49	M	Dairy	No	35	Poor management skills; Chronic malnourishment of herd

H^2^	52	M	Suckler	No	64.2	High mortalities Over stocking, poor quality feed and housing.

I^2^	84	F	Suckler	No	65.8	High mortalities associated with over stocking, poor quality feed and housing conditions

J^2^	42	M	Suckler	No	81.2	Over-stocking; Problems with administration and paperwork

K	31	M	Dairy	No	255.1	High mortality associated with rapid expansion of the herd and poor management skills

L	73	M	Dairy	No	49	High mortality associated with rapid expansion of the herd and poor management skills

M	39	F	Suckler	No	43.5	Poor management skills

N^1,4^	54	M	Suckler	Sheep	339.9	High mortality associated with poor management skills

O^2^	45	M	Suckler	Sheep	42	High mortality

P	57	M	Suckler	Sheep	390	High mortality associated with chronic starvation of the herd; Registration issues

Q	68	F	Suckler	Sheep	44.5	Poor conditions and a lack of food and water; Non-disposal of still-born animals

R	63 & 68	M & F	Suckler	Sheep	116.7	Poor conditions and a lack of food and water; Non-disposal of still-born animals

^1 ^Herd keeper recorded as suffering with ill health

^2 ^Welfare issue recorded as starting after the death of a parent

^3 ^Herd keeper recorded as suffering with depression

^4 ^Herd keeper recorded as having a problem with alcohol

### 3.2 Late registrations

**Figure 1:**
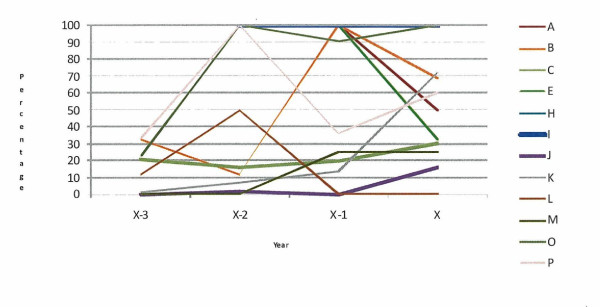
Percentage of animals registered more than 27 days after birth, in the 12 case herds with a defined animal welfare incident (A, B, C, E, H, I, J, K, L, M, O, P), in the incident year (x) and three preceding years (x-3, x-2, x-1)

**Figure 2:**
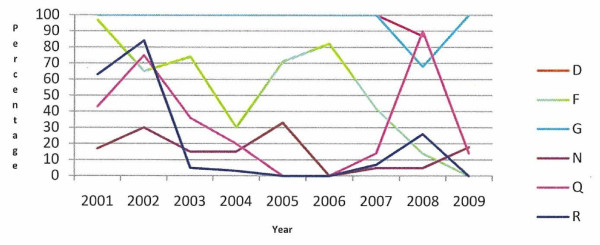
Percentage of animals registered more than 27 days after birth, in the six case herds (D, F, G, N, Q, R) with long-term welfare issues during 2001 to 2009

The percentage of calves registered more than 27 days after birth, in the 12 case herds with defined animal welfare incidents and the 6 case herds with long-term animal welfare issues, is presented in Figures 1 and 2, respectively. All herds with a defined animal welfare incident date registered calves late at some stage over the study period and the majority registered calves late in all years of the study period. All of the long-term herds have registered calves late at some time between 2001 and 2009.

### 3.3 On-farm burial

**Figure 3:**
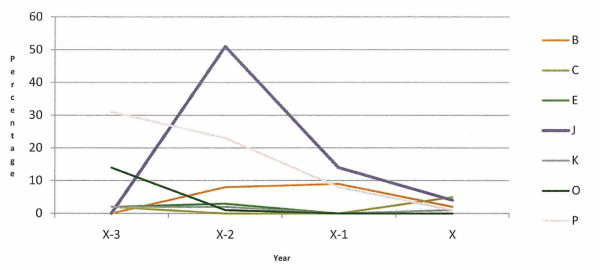
**Percentage of all cattle exits attributed to on-farm burial in seven case herds (B, C, E, J, K, O, P) with a defined animal welfare incident, in the incident (x) and three preceding years (x-3, x-2, x-1)**.

**Figure 4:**
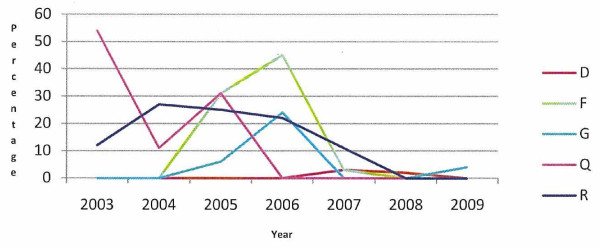
**Percentage of all cattle exits attributed to on-farm burial in five case herds with a long-term animal welfare issue (D, F, G, Q, R), during 2003 to 2009**.

Of the 12 case herds with a defined animal welfare incident, the percentage of all cattle exits attributed to on-farm burial is presented in Figure 3. Five of these herds did not use this method of disposal at any stage either in the incident year or the three preceding years. In five of the six case herds with long-term animal welfare issues, this method of disposal was used at various times after 2002, when it became subject to legislative control and restriction. (Figure 4).

### 3.4 Knackery disposals

**Figure 5:**
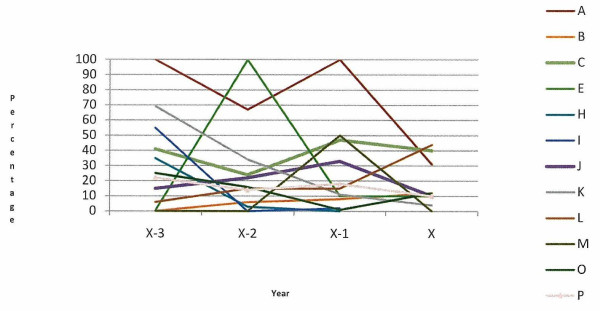
Percentage of all cattle exits attributed to knackery disposal in 12 case herds with a defined animal welfare incident (A, B, C, E, H, I, J, K, L, M, O, P), in the incident and three preceding years

**Figure 6:**
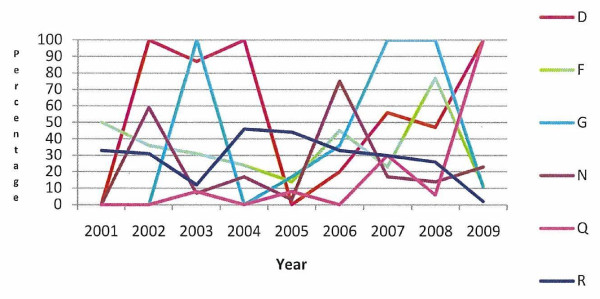
**Percentage of all cattle exits attributed to knackery disposal in six case herds with a long-term animal welfare incident (D, F, G, N, Q, R) during 2001 to 2009**.

Of the 12 case herds with a defined animal welfare incident, the percentage of all cattle exits attributed to knackery disposals is presented in Figure 5. In these herds the number of animals disposed of at knackeries increased annually during the study period, from a median of 5.05% in year x-3 to 21.78% in year x. In the six case herds with long-term animal welfare issues, this figure is presented for the years 2001 to 2009 (Figure 6). All of the long-term herds recorded moves to knackeries in excess of the national average at various stages between 2001 and 2009.

### 3.5 Unaccounted exits

**Figure 7:**
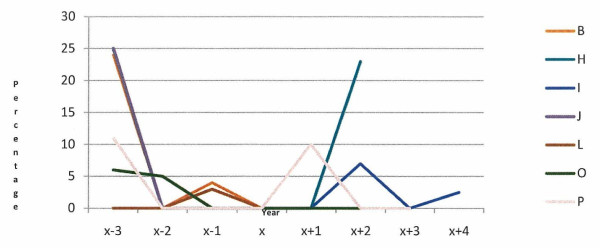
**Unaccounted exits as a percentage of all recorded exits in 7 case herds with defined animal welfare issues (B, H, I, J, L, O, P) during the incident and three preceding years**. In the remaining 5 case herds with a defined animal welfare incident, no such exits were recorded during this period

**Figure 8:**
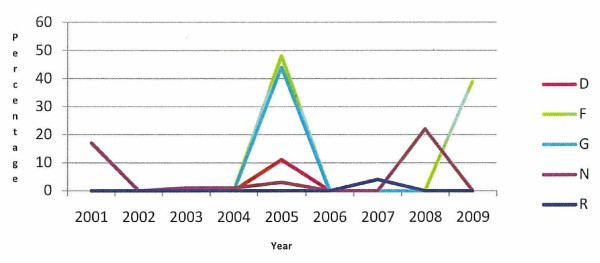
**Unaccounted exits as a percentage of all recorded exits in five case herds with long-term animal welfare issues (D, F, G, N, R) during 2001 to 2009**. On the remaining farm with long-term animal welfare issues, no such exits were recorded during this period

In seven of the 12 case herds with a defined animal welfare incident, unaccounted exits (recorded moves to a 'herd unknown' location) were recorded during the incident and/or three preceding years (Figure 7). Between 2001 and 2009, unaccounted exits were recorded in five of the six case herds with a long-term animal welfare issue (Figure 8).

### 3.6 Changes in herd size

In the 12 case herds with defined animal welfare incidents, the percentage change in the total number of cattle between year x and year x-3 is presented in Table 2. Cattle numbers decreased in five herds, whilst numbers increased in seven herds.

**Table 2:** Percentage change in the total number of cattle between the incident year (x) and the year three years previous (x-3) in 12 case herds (A, B, C, E, H, I, J, K, L, M, O, P) with a defined animal welfare incident date

Herd ID	Number of cattle in year (x-3)	Number of cattle in year (x)	Change (%)
A	9	13	+44.4

B	162	94	-42

C	160	153	-4.4

E	55	37	-32.7

H	162	206	+27.2

I	147	131	-10.9

J	107	106	-0.9

K	105	324	+208.6

L	69	111	+60.9

M	11	14	+27.3

O	97	213	+119.6

P	206	256	+24.3

### 3.7 Numbers of calves registered per cow per year

The number of cows in each herd with a defined animal welfare incident was calculated for consecutive time periods: the incident year and each of the three preceding years. The percentage of available cows that gave birth to a calf that was subsequently registered was determined for this time period (Table 3). No pattern was observed in changes in the number of calves registered per cow per year between the case study herds.

**Table 3:** Percentage of cows in herds in 12 case herds with a defined animal welfare incident date (A, B, C, E, H, I, J, K, L, M, O, P) that gave birth to a calf which was subsequently registered in the incident year (x) and each of the three years leading up to the incident year (x-3, x-2, x-1)

Herd	Percentage of cows that gave birth to a calf that was subsequently registered in each year (number of calves)			
	
	x-3	x-2	x-1	x
A	44.4 (4)	25 (2)	33.3 (2)	50 (4)

B	65.2 (30)	40 (16)	22.5 (9)	82.9 (29)

C	59.8 (52)	50 (50)	62.2 (74)	64.9 (61)

E	35.7 (5)	43.7 (7)	88.9 (16)	27.3 (3)

H	254.8 (79)	108.8 (62)	68.8 (53)	65.3 (64)

I	169 (49)	108.3 (39)	89.6 (44)	67.2 (41)

J	88.1 (37)	77.3 (34)	74.5 (35)	53.2 (25)

K	97.8 (89)	95 (96)	47.1 (65)	28. (81)

L^1^	^1^(8)	66.7 (8)	50 (9)	0 (0)

M	0 (0)	100 (3)	100 (4)	66.7 (4)

O	100 (31)	86.5 (32)	115 (46)	52.5 (31)

P	65.9 (56)	35.5 (33)	119.6 (61)	50 (33)

^1^In herd L there were no females aged 30 months or older registered in the herd on January 1^st ^of year x-3.

## Discussion

The objectives of this current study were to characterize the types of farms investigated by DAFF for animal welfare incidents, using a case study approach and secondly to use these data to enhance EWS. The EWS is a partnership between interested parties who wish to identify ways in which farm animal welfare can be further improved and to identify and resolve animal welfare problems before they become overpowering. It is hoped that by better understanding these case farms we will develop a better understanding as to why, despite the EWS already being in existence, welfare problems still occur on some Irish farms. By studying KPIs we hope to be able to ascertain ways in which to enhance the EWS to make it more effective. This study will form part of a broader study which will include comparisons with control herds and a study of the human factors that influence farm animal welfare incidents.

The welfare of farm animals is well provided for under Irish legislation and on-farm welfare incidents are not common. Traditionally, in Ireland, there has been a perception that welfare problems arise more commonly on farms run by older bachelors living in rural areas, where there is a lack of family support and poor access to health and social facilities. However, in this study the herd keepers ranged in age from young to old (31 to 84 years) and in sex (14 males and 5 females). It is likely therefore that where welfare problems arise, that the underlying cause is not a singular trigger, but may possibly relate to sociological, health, psychological, economic and other circumstances. In five of the case herds the responding SVI/VI believed that problems started to occur after the death of a parent. At the time of the incident two herd keepers were suffering from ill health, one of whom also had reported problems related to alcohol, and another who was reported to be suffering from depression (Table 1). The SVIs/VIs dealing with each welfare incident reported problems on the case farms that included high mortalities, poor management skills, registration issues, carcases unburied on the farm and a prior history of welfare problems. This tallies with the earlier unpublished DAFF study where on the 494 farms investigated 1552 dead animals were found, 78 animals were in extremis and were euthanased on-farm and 17% had issues with tagging and registration (Pat Flanagan: An investigation into On-Farm Welfare Incidents, 2007) and with a study by Collins et al [3] on equine welfare on an Irish farm where abandoned carcases were found, injuries were untreated and there was a history of prior welfare problems.

Our findings indicate that the late registration of calves; the use of on-farm burial as a method of carcase disposal; an increasing number of moves to knackeries over time and records of animals moved to 'herd unknown' were notable on the case farms and warrant further investigation. In contrast, no pattern was established between changes in herd size and the number of calves registered per cow per year between the case study herds (Tables 2 and 3).

Under Regulation EC 1760/2000, all calves must be registered within 27 days of birth. A DAFF communication (DAFF, unpublished data) on all calf registrations in Ireland in 2010 (including these case herds) showed that 2.22% of all 116,815 Irish herd keepers registered calves in excess of the 27 day limit. This figure is similar to the 2.26% of Irish farmers who despite being entitled to receive payments of greater than €100 from the Single Farm Payment Scheme did not apply for these payments (DAFF, unpublished data), possibly indicating problems with completing the paperwork involved. In this study the majority of those herds with a defined animal welfare incident date registered calves late in the incident year and in the years leading up to the incident (Figure 1). All of the long-term herds have registered calves late at some time between 2001 and 2009 (Figure 2). In recent years there has been an increase in the amount of paperwork that herd keepers are required to complete. Studies by Lobley et al. [4], Raine [5], Simkin et al. [6], Booth et al. [7], McGregor et al. [8] and Deary et al. [9] have identified problems with paperwork, legislation and finances as a cause of stress in farmers. Stress can affect decision making and the ability to cope, and may in the current context manifest as failure to manage the herd appropriately. The increased administrative burden may be greater for those farmers with jobs off-farm or with no formal training in agriculture. We do not know if the herd keepers in this study had off farm employment, what level of formal education they received, what their economic status was or what family and community supports were in place.

On-farm burial is prohibited in Ireland, except by licence under EU regulation 1774/2002. In the case study herds with defined animal welfare incident dates, seven herds (58.3%) buried animals on farm during the study period (Figure 3). Only one of the six herds, where the incident was determined to be long-term, did not bury animals on-farm after 2002 (Figure 4). In April 2009, DAFF ceased to make financial contributions to the Fallen Animals Scheme, except for animals aged 48 months or older. This scheme helped with the cost of disposing of animals through knackeries. As the most recent welfare incident date from the case herds was April 2009, it is unlikely that the abolition of the subsidy was a reason for the on-farm burials recorded in this study. On-farm burials may indicate that herd keeper is trying to conceal a welfare problem or that he/she is ignorant of, or showing a disregard for, the legal requirements of animal disposal. A further study will compare on-farm burial in these herds with control herds.

A knackery is a plant in which unprocessed Category 2 material (material unfit for human consumption) is handled and/or temporarily stored for the purpose of further transportation to its final destination. Between 2002 and 2009 the total number of cattle on-farm deaths in Ireland ranged from 192,437 to 234,088, which equates to 9.1% to 10.9% of the total number of cattle disposals, with a peak in 2008 at 12.1% and a low in 2003 of 8.6% (DAFF, unpublished data). For the 12 case herds with a defined animal welfare incident date, the number of animals disposed of at knackeries in relation to all exits from the farm increased annually during the study period, from a median of 5.1% three years prior to the incident, 14.6% two years prior to the incident, 16.8% in the year prior to the incident and 21.8% in the year that the incident occurred (Figure 5). The high median in the year the incident occurred is likely to be due in part to DAFF intervention in welfare cases where burial on farm was not allowed as a method of disposal of carcases already present on farm and to the euthanasia of animals too ill to move off the farm. The increase in the number of on-farm deaths of animals in the study in the two years preceding the incident year suggests that there was an increase in the numbers of animals that were not fit to enter the human food chain that either died on-farm or were euthanased on farm. This is likely due to disease, poor condition and unfitness to travel. All of the long-term herds recorded moves to knackeries in excess of the national average at various stages between 2001 and 2009 (Figure 6).

Another of our findings that was notable was moves to 'herd unknown' location. A 'herd unknown' location is used to record situations where the final end of life point of the animal cannot be determined with absolute certainty. In 2010, there were several cases of cattle rustling reported in Ireland [10]. It is believed that some animals that die on-farm from welfare and disease issues are buried on-farm and subsequently reported as stolen or lost by the herd keeper. Cattle herds are subject to an annual Tuberculin test in Ireland. At the time of test, herd profile data including the location and registration details of the animals due to be presented at the TB test in each herd, as recorded on AIM, can be downloaded by the testing veterinarian from AHCS. Any discrepancies found are flagged on the system and subjected to intensive systematic investigations by DAFF. Whilst most of the discrepancies are resolved through this process, if the discrepancy cannot be resolved with certainty, the tag number is listed in 'herd unknown' location. In this study, the location of various animals in seven of the 12 herds where the incident date was known was unable to be resolved (Figure 7). Only one of the long-term herds did not record any moves to this location in the years 2001 to 2009 (Figure 8). The high percentage of the case study herds with moves to 'unknown location' during the three years leading up to a welfare incident indicates that moves to this location are of concern, however it is likely that these animals were assigned retrospectively to 'herd unknown' location in the years preceding the incident after an investigation was completed. Attention should be paid to discrepancies that cannot be resolved within a timely manner.

In 2009 (the year that this study commenced), there were 117,287 herds in Ireland. The average age of farmers was 55.8 years [11]. The most current information on average farm size is for 2007, where it was 32.3 hectares [12]. We were surprised at the diversity of the herd keepers in this study with respect to age and sex, and with the range in sizes of the farms (Table 1). Some factors such as age tallied with the national average, but others, e.g. average farm size, did not.

Socio-economic factors are likely to play a key role in animal welfare incidents e.g. family support; changes in personal circumstances etc., as welfare problems are not always due to intentional neglect or cruelty by herd keepers. A study by Sanne et al. [13] identified that male agricultural workers had the highest level of depression of all occupational groups and that the level of anxiety in male farmers was significantly higher than the average level among all working male participants. Due to long working hours and distance from mental health care facilities many herd keepers experiencing mental health issues may not get help. During the Celtic Tiger years, off-farm employment of either the farmer and/or spouse rose year on year from 45% of farms in 2001 to a peak of 58% in 2006 and 2007 [14]. This should be of concern given the extent to which off-farm income supports the viability of many farms, leading to many herd keepers not having sufficient time to devote to their livestock. This can give rise to welfare problems.

There are limitations with this study, including the small number of case study herds. The data for KPIs were all taken from national databases, which rely on herd keepers providing the correct information. There is a potential for bias in the case studies chosen as they were nominated by SVIs and not selected randomly. In addition, this study did not evaluate other factors such rural isolation, availability of family and community support, accessibility of health care and off-farm employment, which may also be important key indicators. This will be addressed in further studies, as will comparisons with control herds.

## Conclusion

This study was carried out to describe aspects of case study herds investigated by DAFF in which animal welfare incidents occurred and to identify key performance indicators (KPIs) that can be monitored to enhance the EWS. Six KPIs were identified, four of which could be used to enhance the early warning system already in place in Ireland, i.e. late registrations of calves, an increase in the use of on-farm burial as a method of carcase disposal, an increase in the number of carcases sent to knackeries and animals missing from the herd profile that cannot be accounted for. Further investigation by studying these KPIs in control herds is warranted to determine their potential to provide a framework for refining current systems of early warning and prevention.

## Competing interests

The authors declare that they have no competing interests.

## Authors' contributions

PK provided overall leadership to the study, and conducted the data collection and analysis. All authors contributed to the study design and participated in writing the paper. All authors have read and approved the final manuscript.
